# Effects of a single subanesthetic dose of esketamine on postoperative subthreshold depressive symptoms in patients undergoing unilateral modified radical mastectomy: a randomised, controlled, double-blind trial

**DOI:** 10.1186/s12888-024-05753-9

**Published:** 2024-04-24

**Authors:** Huanwei Wang, Rigen Te, Jianxing Zhang, Yanbing Su, Hongxia Zhou, Na Guo, Dongmei Chi, Wan Huang

**Affiliations:** State Key Laboratory of Oncology in South China, Guangdong Provincial Clinical Research Center for Cancer, Sun Yat-sen University Cancer Center, 651 Dongfeng East Road, Guangzhou, 510060 People’s Republic of China

**Keywords:** Esketamine, Breast Cancer, Postoperative Subthreshold Depressive Symptoms

## Abstract

**Background:**

Breast cancer is the most common malignant tumor in females worldwide. During disease development, breast cancer patients suffer anxious and depressed, which may lead to worse quality of life or even higher mortality. Esketamine has been regarded as an antidepressant in breast cancer patients with mild or moderate depression. Here, we wonder whether the administration of esketamine could reduce the postoperative depressive symptom score of breast cancer patients who have no preoperative depression.

**Methods:**

A total of 64 patients treated with unilateral modified radical mastectomy were randomly divided into an experimental group (esketamine group, Group E) and a control group (Group C), with 32 cases in each one. After anesthesia induction, Group C received 0.2 ml/kg of normal saline intravenously and Group E was administered 0.2 mg/kg intravenous esketamine. The primary outcome was the Patient Health Questionnaire-9 (PHQ-9) scores. The secondary outcomes included the Visual Analogue Scale (VAS) scores for pain, inflammatory markers, perioperative-related indicators, and the incidence of postoperative delirium, nausea and vomiting.

**Results:**

The PHQ-9 score on postoperative day (POD) 1 in Group E declined from the preoperative level, while the score in Group C was higher than before, and the former was far lower than the latter (*P* = 0.047). There is no statistically significant difference in PHQ-9 scores between Group E and Group C on POD 3, 7, and 30. Moreover, the postoperative leukocyte level of Group E was higher than that of Group C, and the difference was statistically significant (*P* = 0.030).

**Conclusions:**

A single subanesthetic dose of esketamine can result in lower postoperative score on subthreshold depressive symptoms compared to the Group C on POD 1, without increasing the occurrence of postoperative adverse reactions.

**Trial registration:**

Registration number: Chinese Clinical Trial Registry ChiCTR2200057028. Date of registration: 26/02/2022.

## Background

Breast cancer is the most common malignant tumor in females worldwide. Based on the 2020 global tumor epidemiological statistics, breast cancer had an annual diagnosis of approximately 2.3 million cases worldwide. This number exceeds the incidence of lung cancer and accounts for 11.7% of all cases of malignant tumors [1]. During disease development, breast cancer patients feel anxious and depressed oftentimes alongside their body symptoms related to the cancer [2]. In a study conducted by So WK et al., 218 breast cancer patients were examined, revealing a prevalence of 21.1% for anxiety, 34.4% for depression, and 15.6% for comorbidity between anxiety and depression [3]. Beyond health issues, anxiety and depression may make it hard for patients to engage in family or work, leading to an even worse quality of life [4]. Additionally, patients with cancer, if experience both anxiety and depression, their mortality is 19% higher than that of patients only suffering from cancer [5]. Though postoperative depression is well-studied, its prevention and treatments are still clinical puzzles to be solved [6].

Ketamine is an N-methyl-D-aspartate (NMDA) receptor antagonist acting in the central nervous system, it helps calm, relieve anxiety, and ease pain [7–9]. Besides, ketamine can be used for treatment-resistant depression (TRD) [10]. Esketamine is the dextrorotatory molecule of ketamine. Compared with ketamine, esketamine, at half the ketamine dosage, relieves pain better and has a higher clearance rate with fewer side effects [11]. Esketamine has been used as an antidepressant in recent years and some articles said that esketamine can stably alleviate depression in adult TRD patients [12]. A single dose of ketamine can rapidly increase the release of presynaptic glutamate and enhance regional activity in the excitatory network by antagonizing NMDA receptors [13, 14]. This process ultimately leads to significant alterations in synaptic plasticity and connectivity, resulting in a rapid antidepressant effect [15, 16]. Its anti-depression effect does not fully count on NMDA receptor antagonists and may involve multiple substances and mechanisms [17, 18].

According to some studies, a subanesthetic dose of esketamine is more effective than ketamine against postoperative depression in breast cancer patients with mild or moderate depression before surgery [19]. Currently, we still do not know if the use of esketamine can reduce the postoperative depressive symptom score of breast cancer patients who have no preoperative depression. This trial probes into the effects of a single subanesthetic dose of esketamine on patients’ postoperative depressive symptom score after unilateral modified radical mastectomy. We hypothesized that the administration of esketamine could reduce the score of breast cancer patients. In addition, Wencai Tu et al. confirmed that the use of 0.5 mg/kg esketamine at the induction of anesthesia lessened the perioperative inflammatory response in elderly surgical patients [20]. Thus, we also assume that the use of esketamine can reduce postoperative Visual Analogue Scale (VAS) scores for pain and has anti-inflammatory effects.

## Methods

This trial was conducted in Sun Yat-sen University Cancer Center and the aim of it is to explore the effects of a single subanesthetic dose of esketamine on postoperative depressive symptom score in patients following unilateral modified radical mastectomy.

### Patients

64 patients that have undergone unilateral modified radical mastectomy between May 2022 and March 2023 were included in the research and the following inclusion criteria were applied:

1. 18 to 55 years of age
2. American Society of Anesthesiologists (ASA) Classification: ASA I or ASA II
3. Preoperative pathology reports of breast cancer and planned unilateral modified radical mastectomy

However, the following patients will be excluded:

1. The patients have esketamine contraindications
2. The patients have experienced depression treatments in the past 2 months
3. The patients have a medical history of mental disorders or severe systematic diseases including heart, liver, or kidney diseases

### Analgesia strategies

Subject patients will be divided into two groups randomly:

- 1) The control group (Group C): participants received an intravenous infusion of 0.2 ml/kg normal saline after anesthesia induction.
- 2) The esketamine group (Group E): participants received an intravenous infusion of 0.2 mg/kg esketamine (Jiangsu Hengrui Pharmaceutical Co., Ltd, Lianyungang City, Jiangsu Province, China) after anesthesia induction.

Patients’ electrocardiogram, heart rate, blood oxygen saturation, non-invasive blood pressure, and depth of anesthesia were monitored regularly before anesthesia. Then, 3 ng/ml remifentanil, 0.3 mg/kg etomidate, and 0.2 mg/kg cisatracurium were injected intravenously with target controlled infusion (TCI) pumps before endotracheal intubation and mechanical ventilation. Anesthesia was maintained with 2–2.5% inhaled sevoflurane anesthetics, 0.5–3 ng/ml remifentanil, and 1.5 ug · kg^−1^ · min^−1^ cis-atracurium; sevoflurane concentration was adjusted based on the depth of anesthesia. After surgery, a 50 mg flurbiprofen was given intravenously, and 5 mg dexamethasone and 12.5 mg dolasetron were prepared to prevent postoperative nausea and vomiting. If the patient requests additional analgesics after surgery, we will inject 50 mg of tramadol intravenously.

### Outcomes

The primary outcome was the Patient Health Questionnaire-9 (PHQ-9) scores. The secondary outcomes included the VAS scores for pain, inflammatory markers, perioperative-related indicators, and the incidence of postoperative delirium, nausea and vomiting. PHQ-9 was adopted as the self-assessment scale to check the severity of depressive symptoms. It includes 9 evaluation items, each of which is scored 0–3 points based on severity. The range of PHQ-9 score is 0–27 points (0–4, Normal or no signs of depression; 5–9, Mild depression; 10–14, Moderate depression; 15–19, Moderate severe depression; 20–27, Severe depression). They were collected on postoperative day (POD) 1, 3, 7, and 30 respectively before pain assessment with VAS. The VAS score ranges from 0 to 10 points based on the severity of pain (0, No pain; 1–3, Mild pain; 4–6, Moderate pain; 7–10, Severe pain). Patients’ baseline data (age, height, weight, basic diseases history, and surgical history), surgical situation (anesthesia time, remifentanil dosage, extubation time, and postoperative delirium, nausea and vomiting), leukocyte level and C-reactive protein (CRP) levels (1 day before and after surgery) were gathered as well.

### Randomization and blinding

This is a randomised, controlled, double-blind trial. 64 patients were divided randomly into two groups (32 patients in each group) by the trial research randomization system in the Sun Yat-sen University Cancer Center. Independent third-party researchers were responsible for the random grouping and the preparation of medicine to be used after anesthesia induction without informing patients, anesthesiologists, and other staff in the research team of relevant information.

### Statistical analysis

To examine the effects of the intervention, 6 pre-experiments were conducted, consisting of 3 cases in Group C and 3 cases in Group E. The primary outcome was the PHQ-9 score on POD 1, which yielded a score of 6.33 for Group C and 3.33 for Group E. To determine the sample size, a one-tailed test was specified with α = 0.025 and a test power of 1-β = 0.9. Using PASS version 15.0 for calculation, it was determined that 25 cases were required for each group. Taking into account a potential dropout rate of 20%, a total of 32 cases were needed for each group, resulting in a total of 64 cases for the experiment.

Quantitative data were described by mean ± SD, and the qualitative data were described by frequencies. Independent-Samples T-test was used to compare the quantitative data of Group C and Group E, and the Shapiro–Wilk test was used for the normality test. Mann–Whitney U test would be adopted when the data did not follow the normal distribution. Comparison of qualitative data of the two groups were done through the Chi-Square test or Fisher’s Exact test. Statistical analysis was performed with SPSS version 25.0 (IBM Corp., Armonk, NY, USA) and GraphPad version 8.0 (GraphPad Software, San Diego, CA, USA). All statistical tests were two-tailed tests, and *P* < 0.05 was considered statistically significant.

## Results

### Baseline data

**Fig. 1:**
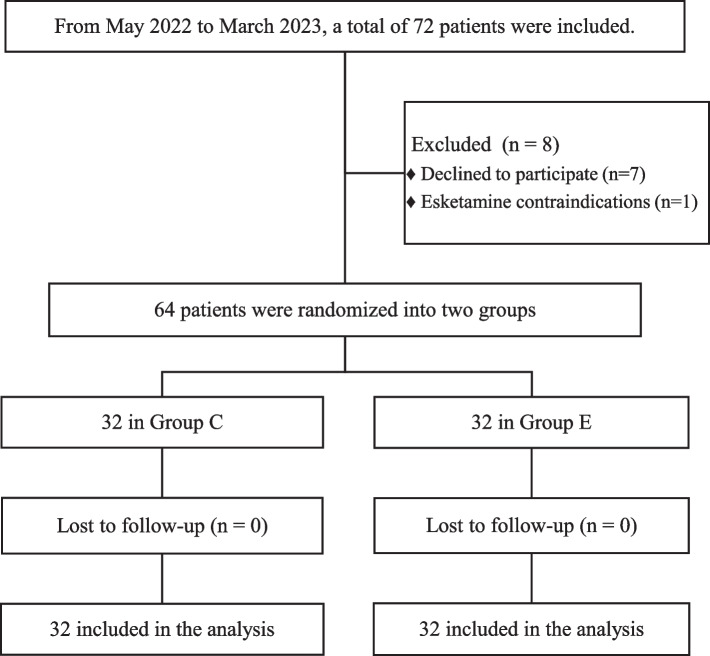
The flowchart of patient participanting in the study. Seven patients were excluded due to refusal to participate, one was excluded because of esketamine contraindications. Group C, control group. Group E, esketamine group

We analyzed the data of 64 patients (Fig. 1). This trial included 32 patients (median age, 42.3 ± 5.8 years) in Group E and 32 patients (median age, 41.8 ± 5.8 years) in Group C (Table 1). As patients of both groups had no significant contrast in age, body mass index (BMI), history of basic diseases, or surgical history, Group E and C were comparable. During the research, no participants were absent for follow-ups.

**Table 1:** Basic data of patients in the two groups

	Control group	Esketamine group	*P*-value
Sample size	32	32	
Age (year)	41.8 ± 5.8	42.3 ± 5.8	0.671
Height (cm)	158.2 ± 6.1	159.2 ± 5.5	0.493
Weight (kg)	55.8 ± 8.0	58.6 ± 10.3	0.460
BMI (kg/m^2^)	22.4 ± 3.3	23.1 ± 3.6	0.393
Basic diseases (n)			
Hypertension	1	2	1.000
Diabetes	1	1	1.000
Others	3	3	1.000
Surgical history			
Yes (n)	12	12	1.000

*BMI* body mass index

### Perioperative Situations and Inflammation Markers

**Fig. 2:**
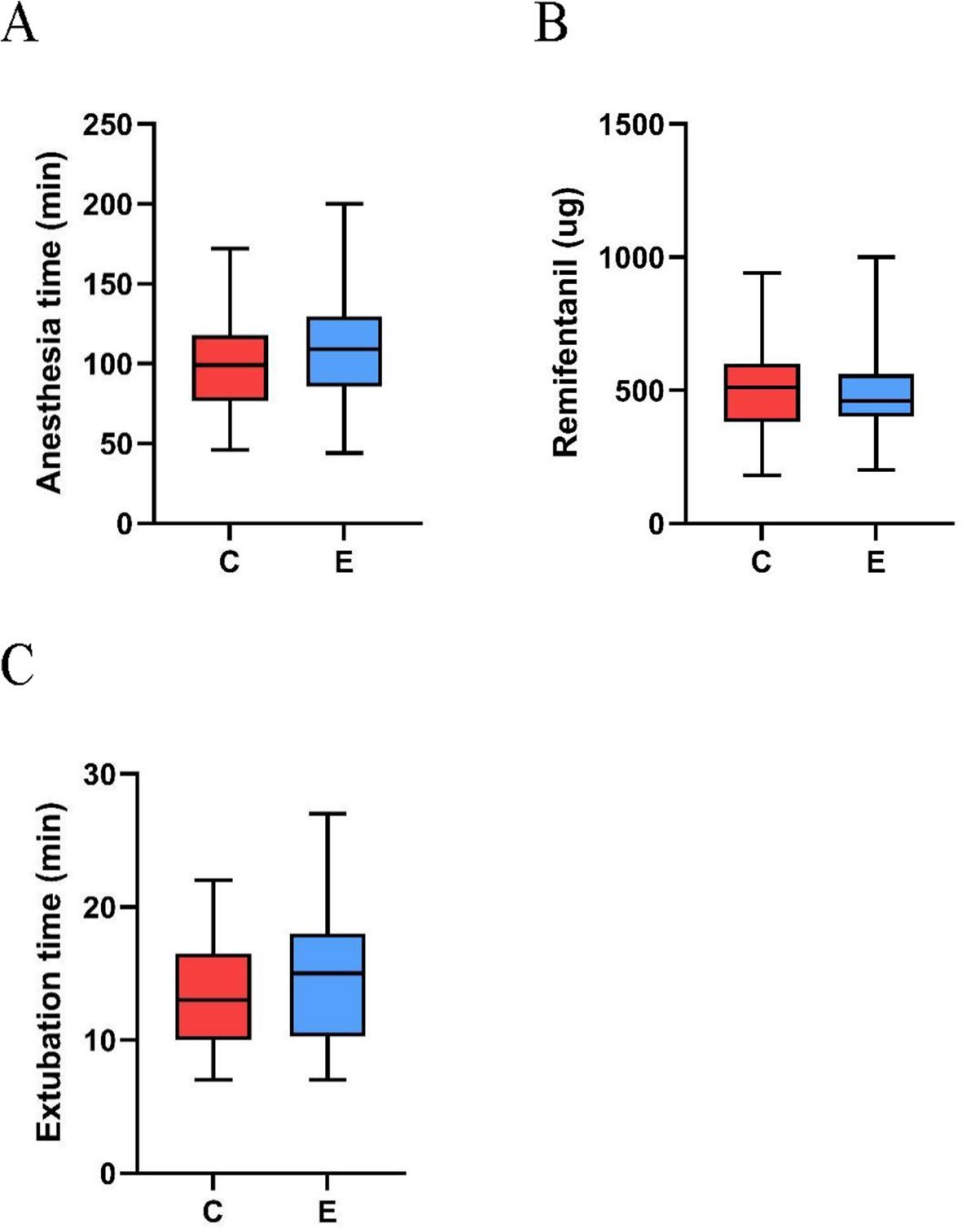
Perioperative related indicators of two groups. **A** The anesthesia time in two groups; (**B**) the dosage of remifentanil in two groups; (**C**) the extubation time in two groups. The box plot demonstrates the maximum, minimum, median, and quartile of various perioperative related indicators. C, control group. E, esketamine group

**Fig. 3:**
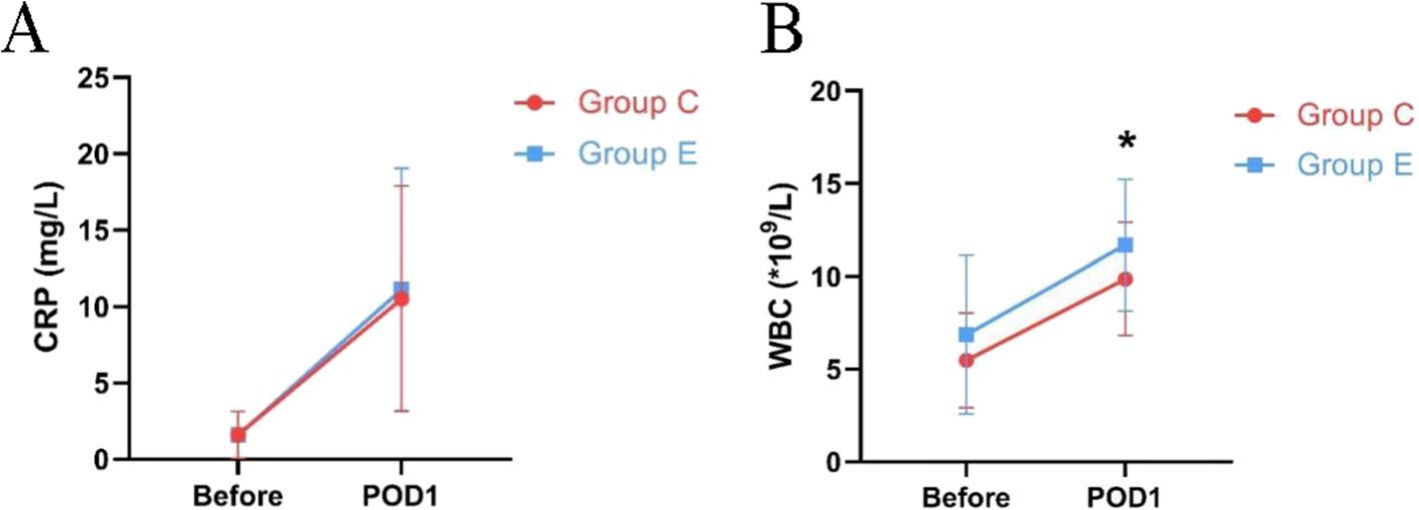
Changes in preoperative and postoperative inflammatory markers of two groups. (**A**) The CRP in two groups; (**B**) the WBC in two groups. The graph displays the mean and standard deviation of inflammatory markers collected preoperatively and on POD 1 in Group E and C, the Independent Samples T-test was used for statistical analysis. Group C, control group. Group E, esketamine group. CRP, C-reactive protein. POD, postoperative day. WBC, leukocyte. Compared with the control group at the same time point, *P < 0.05

There was no statistical significance in anesthesia time, intraoperative remifentanil dosage, extubation time, and postoperative delirium, nausea and vomiting between the two groups (Table 2 and Fig. 2). This meant the use of esketamine did not prolong extubation time or increase the incidence of postoperative adverse reactions. There was no statistically significant difference in both preoperative and postoperative CRP levels between Group E and Group C. There was no statistically significant difference in leukocyte levels between the two groups preoperatively (*P* = 0.094). But the postoperative leukocyte level of Group E was higher than that of Group C, and the difference was statistically significant, the Independent Samples T-test was used for statistical analysis. (*P* = 0.030) (Fig. 3). This means that in this trial, esketamine exhibited a pro-inflammatory effect.

**Table 2:** Relevant data and inflammatory markers of patients during perioperation

	Control group	Esketamine group	*P*-value
Anesthesia time (min)	102.6 ± 32.9	106.0 ± 31.9	0.678
Extubation time (min)	13.5 ± 4.2	14.9 ± 5.1	0.239
Remifentanil (ug)	513.0 ± 179.9	489.9 ± 175.6	0.519
Complication (n)			
Nausea	6	6	1.000
Vomit	3	2	1.000
Delirium	0	0	1.000
CRP (mg/L)			
Before	1.60 ± 1.52	1.58 ± 1.53	0.813
POD 1	10.51 ± 7.38	11.12 ± 7.93	0.787
WBC (*10^9^/L)			
Before	5.47 ± 2.55	6.85 ± 4.28	0.094
POD 1	9.86 ± 3.05	11.69 ± 3.54	0.030

*CRP* C-reactive protein. *POD* postoperative day. *WBC* leukocyte

### PHQ-9 and VAS scores

**Fig. 4:**
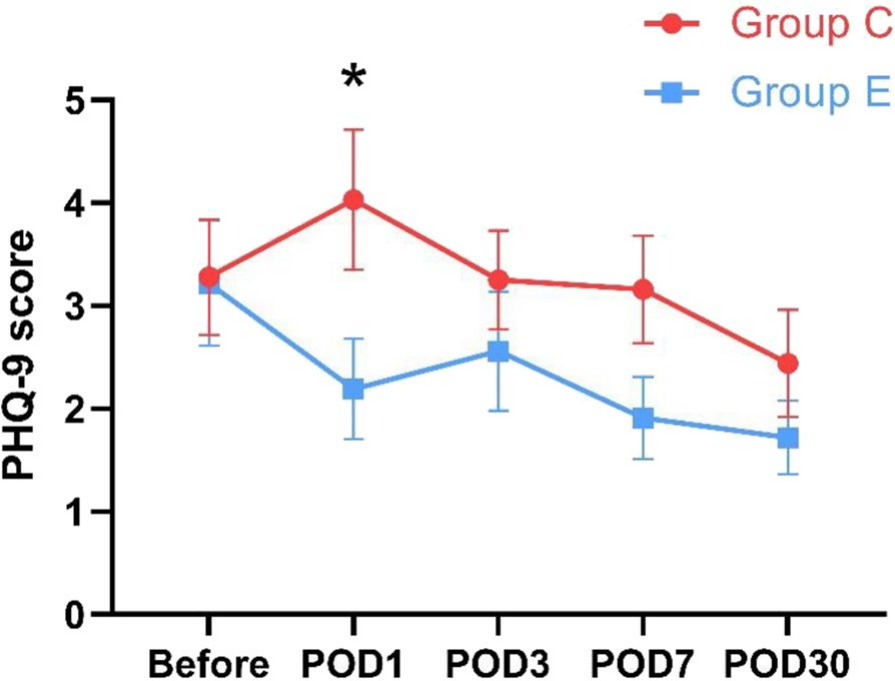
Changes in preoperative and postoperative PHQ-9 scores between the two groups. The graph shows the mean and standard error of the perioperative PHQ-9 scores of the two groups, and scores of Group E and C at the same time point was compared with the Mann–Whitney U test. Group C, control group. Group E, esketamine group. PHQ-9, Patient Health Questionnaire-9. POD, postoperative day. Compared with the control group at the same time point, *P < 0.05

**Fig. 5:**
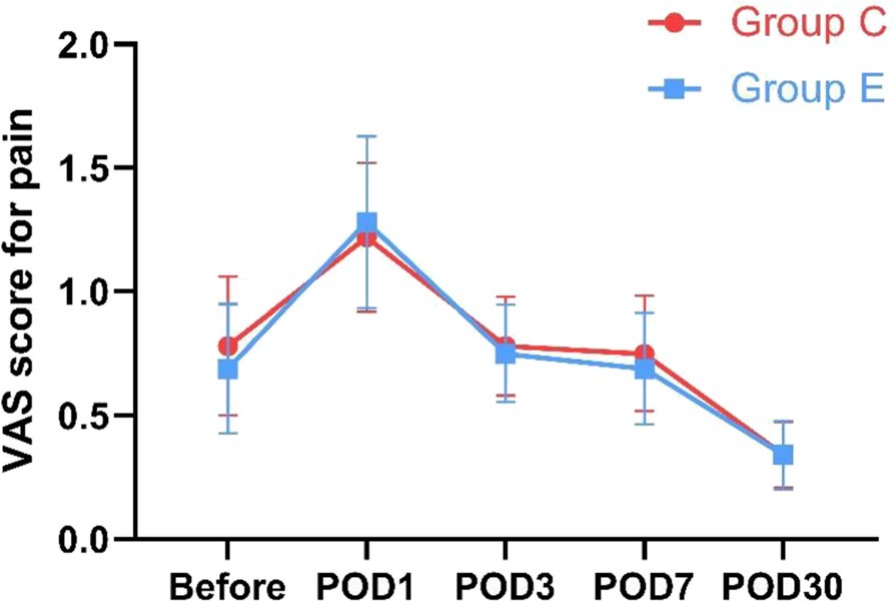
Changes in preoperative and postoperative VAS scores for pain of two groups. The graph shows the mean and standard error of the perioperative VAS scores of Group E and C. Group C, control group. Group E, esketamine group. VAS, visual analogue scale. POD, postoperative day

Scores of Group E and C at the same time point was compared with the Mann–Whitney U test. There was no statistically significant difference in the PHQ-9 scores of the two groups preoperatively (*P* = 0.718) (Table 3). The PHQ-9 scores of Group E on POD 1 decreased while the scores of Group C increased after surgery, and the former was significantly lower than the latter (*P* = 0.047). There was no statistically significant difference in PHQ-9 scores between Group E and Group C on POD 3, 7, and 30 (Fig. 4). This show that a single administration of esketamine result in lower postoperative depressive symptom score compared to the Group C on POD 1. The VAS scores for pain of both groups saw a remarkable increase on POD 1, but there was no statistically significant difference in both preoperative and postoperative VAS scores between the two groups (Fig. 5). There were 2 cases in each group requiring additional analgesics after surgery, and all cases were treated with 50 mg tramadol. This show that better postoperative analgesic effect was not observed in Group E.

**Table 3:** Preoperative and Postoperative PHQ-9 and VAS scores of two groups

	Control group	Esketamine group	*P*-value
PHQ-9 score			
Before	3.28 ± 3.15	3.22 ± 3.44	0.718
POD 1	4.03 ± 3.84	2.19 ± 2.75	0.047
POD 3	3.25 ± 2.71	2.56 ± 3.27	0.143
POD 7	3.16 ± 2.94	1.91 ± 2.26	0.100
POD 30	2.44 ± 2.94	1.72 ± 2.05	0.482
VAS score			
Before	0.78 ± 1.58	0.69 ± 1.47	1.000
POD 1	1.22 ± 1.70	1.28 ± 1.97	0.996
POD 3	0.78 ± 1.13	0.75 ± 1.11	0.951
POD 7	0.75 ± 1.32	0.69 ± 1.28	0.994
POD 30	0.34 ± 0.75	0.34 ± 0.79	1.000

*PHQ-9* Patient Health Questionnaire-9. *POD* postoperative day. *VAS* visual analogue scale

## Discussion

In this trial, we discovered that a single dose of esketamine can result in lower postoperative depressive symptom score compared to the Group C on POD 1, without causing an increase in postoperative adverse reactions. However, we did not observe any improved postoperative analgesic or anti-inflammatory effects in Group E, which received esketamine.

Over the past years, there has been much research on the effects of ketamine or esketamine on postoperative depression in patients undergoing different surgery. Cheol Lee and others found that a single dose of ketamine after general anesthesia induction can significantly improve depression scores on POD 1 in patients treated with gynecologic laparoscopic surgery [21]. Besides, according to a study of 670 elderly patients by G. A. Mashour et, al., intraoperative use of a subanesthetic dose of ketamine seemed not to prevent or improve depressive symptoms on POD 3 and 30 in elderly patients that experienced major surgery [22]. These findings align with the results of this trial. However, Min Jiang et al. conducted a study that showed the intraoperative use of ketamine to be effective in alleviating depression on POD 1 and 5 in patients undergoing elective orthopedic surgery. The antidepressant effect of ketamine lasted until POD 5, which could be attributed to the continuous infusion of ketamine at a rate of 0.25 mg · kg^−1^ · h^−1^ for 30 min, in addition to the administration of a 0.5 mg/kg ketamine dose during anesthesia induction [23]. In this trial, the administration of esketamine differed from the previous study. Instead of a continuous infusion, esketamine was given as a single dose. This difference in dosing method may potentially affect the duration of the antidepressant effect of esketamine.

Jie Wang et al. found that in cervical cancer patients who underwent total laparoscopic hysterectomies and had preoperative mild to moderate depression, the use of a subanesthetic dose of esketamine during the surgery resulted in a decrease in depression scores on POD 3. This effect was more significant when compared to the same dose of ketamine [24]. Peirong Liu et al. also discovered that a single administration of esketamine after induction of anesthesia greatly eased postoperative depression in breast cancer patients with preoperative mild to moderate depression, and its effect of esketamine continued to exist till 1 month after surgery [19]. The contrasts in the findings between the studies mentioned above and this trial may attribute to different research subjects. Previous studies focused on evaluating the effectiveness of esketamine in patients with preoperative depression, whereas this trial specifically examined the impact of esketamine on postoperative depressive symptom score in patients without preoperative mental disorders. In this trial, the PHQ-9 scores reveal that the subjects predominantly displayed subthreshold depressive symptoms after undergoing surgery. This indicates that a single subanesthetic dose of esketamine can result in lower postoperative score on subthreshold depressive symptoms compared to the Group C on POD 1.

Previous research conducted a few years ago discovered that ketamine has the ability to significantly decrease the production of pro-inflammatory cytokines while not interfering with the generation of anti-inflammatory cytokines [25–28]. This suggests that ketamine could be utilized as a means to reduce inflammation. However, no anti-inflammatory effect of esketamine was observed in this trial. On the contrary, the postoperative leukocyte level in Group E was higher than that in Group C. This may be partly explained by the lower dose of esketamine in the research, and the time dependence and dose dependence of ketamine as an anti-inflammatory agent. Recent studies have revealed that ketamine has the ability to promote both anti-inflammatory and pro-inflammatory responses. As a result, it should be seen more as a modulator that facilitates the dynamic balance of the immune system [29]. In addition, as surgery can cause varying degrees of inflammatory response, this may affect the measurement of the effect of esketamine on inflammatory response.

In this trial, no negative effects such as prolonged extubation time, or increased the incidence of postoperative delirium, nausea and vomiting were observed in Group E that used esketamine, and there were no significant differences in remifentanil dosage and VAS scores for pain between the two groups. Better postoperative analgesic effect was not observed in Group E maybe due to the low esketamine dose.

The major limitation of this trial may be the instrument for identifying the severity of depressive symptoms. Relative to a specialized depression questionnaire or a clinical assessment, the adopted self-assessment questionnaire is likely to influence the research results. A multicenter, large sample research is required to determine the optimal usage, dosage, and efficacy of esketamine in reducing postoperative depressive symptom score, as this study only administered a low dose of esketamine. As the high incidence of postoperative depression in breast cancer patients who have received surgery can seriously impact their quality of life and even lead to a rise in mortality, effective preventive interventions are badly needed.

## Conclusions

A single subanesthetic dose of esketamine can result in lower postoperative score on subthreshold depressive symptoms compared to the Group C on POD 1, without increasing the occurrence of postoperative adverse reactions.

## Data Availability

The datasets used and/or analysed during the current study are available from the corresponding author on reasonable request.

## Abbreviations

Group E: Esketamine group
Group C: Control group
PHQ-9: Patient Health Questionnaire-9
VAS: Visual Analogue Scale
POD: Postoperative day
NMDA: N-methyl-D-aspartate
TRD: Treatment-resistant depression
CONSORT: Consolidated Standards of Reporting Trials
ASA: American Society of Anesthesiologists
TCI: Target controlled infusion
CRP: C-reactive protein
WBC: Leukocyte
BMI: Body Mass Index
